# Anticipatory Grief in Dementia: An Ethnographic Study of Loss and Connection

**DOI:** 10.1007/s11013-022-09792-3

**Published:** 2022-06-29

**Authors:** Natashe Lemos Dekker

**Affiliations:** Institute of Cultural Anthropology and Development Sociology, Leiden University, Wassenaarseweg 52, 2333 AK Leiden, The Netherlands

**Keywords:** Dementia, Anticipatory grief, Loss, Relations, Subjectivity

## Abstract

In this article, I address the experiences of family members of people with dementia, as they expressed the sensation of gradually losing the person with dementia. Based on ethnographic fieldwork in nursing homes in the Netherlands, and contributing to the anthropology of grief, I explore the co-existence of experiences of anticipatory grief and manifestations of care to maintain meaningful relations. I show how my interlocutors adapted to changing circumstances as the disease progressed, and in so doing found new ways to relate, as well as prepared for future losses and the expected end of life. I argue that anticipatory grief is temporal and relational, encompassing both present and future losses, and involving a continuous negotiation between the loss and the continuing relationship. I underscore the entanglement of loss and connection, showing how both exist parallel to, and may emerge from one another, and demonstrating how an anthropological approach to anticipatory grief can reveal the nuanced and equivocal character of experiences of illness and at the end of life.

## Introduction

“Often it feels as if I already lost her, as if she is not my mother anymore. It is a process of grief that started a long time ago,” said Patrick, who visited his mother with dementia in the nursing home every Sunday. During the one and a half year I spent doing fieldwork in nursing homes in the Netherlands, family members, like Patrick, often expressed the feeling that they had already lost or partly lost their relative with dementia, actively addressing their experiences as “already grieving” while the person with dementia was still alive. Such experiences resonate with popular discourses and imaginaries in Western societies that frame people with dementia in terms of a loss of life and of the person, and in which both the relationship with the person with dementia and their subjectivity are at risk. However, while the narratives of family members often revolved around experiences of loss, ethnographically, I also saw that they cultivated ways to maintain meaningful relations with the person with dementia. Facing loss, they continuously tried to find what was “still possible” to share meaningful moments and keep the person with dementia connected to the world. Such ambiguity of experiencing loss while at the same time maintaining a relationship, emerged during the trajectory of dementia, when my interlocutors witnessed the cognitive and physical capacities of their relatives diminish, and changes in the relationships between them.

In this article, I explore this co-existence of efforts to maintain meaningful relations and experiences of what has been referred to in the literature as pre-death, or anticipatory grief in dementia (Blandin and Pepin 2015; Large and Slinger 2015; Lindauer and Harvath 2014; Moore, et al., 2020; Peacock, et al., 2018). While public discourses tend to address dementia in terms of loss, using metaphors of absence and disappearance, there is also an increasing number of studies that address the possibilities of a good life with dementia (Driessen 2018; Grøn and Mattingly 2018), the relational, as opposed to biomedical, practices through which the person with dementia is held in relations (Moser 2011), and arts-focused moral experiments to engage the person with dementia (Jeong 2020; Taylor 2017). My contribution to this growing body of literature is to demonstrate the ways in which loss and connection interweave and sometimes come apart. I am interested, in other words, in demonstrating how family members navigate ambiguous attachments by continuously adapting to, and anticipating, changing circumstances. While this recent attention for alternative and more positive perspectives on dementia is valuable and much needed, there is an equally pressing need to understand the experiences of grief that may accompany illness and dementia. Indeed, others have cautioned for the risk of romanticizing dementia when addressing it (only) in terms of “living well” and have called for a nuanced view on the suffering involved, without reinforcing the negative tropes associated with the disease (Grøn forthcoming; Bartlett et al. 2017). Hence, my aim in this article is to acknowledge and create space for ambiguous experiences and seemingly contradictory emotions, narratives, and practices. By focusing on the experiences of grief and loss of family members *as well as* their efforts to maintain meaningful relations, I argue that in caring for a person with dementia, loss and connection are interwoven.

Anthropologists have demonstrated extensively that mourning rituals can be understood as social expressions of grief. In the anthropology of death, many studies have considered the social meaning of funerary rituals, which can be traced back to Hertz (1907), Van Gennep (1909), Radcliffe-Brown (1922), and Huntington and Metcalf (1979) in their delineations of funerary rituals as transitory and transformational. Considering that death causes an interruption in the social order, rituals are seen as restoring this gap in the social body and reintegrating the bereaved into society (Howarth 2007; Robben 2004; Shohet 2018). In addition to this long-standing and continuing interest in death rituals, anthropologists have increasingly sought to understand the emotional aspects of grief. Particularly the work of Rosaldo (1989, 2014), who reflected on the subjectivity of the experience and emotional “force” of grief, signaled a need to study the affective aspects of grief anthropologically. Scheper-Hughes (1993), in turn, has shown how the experience of grief is also subject to politics and structural inequalities. These works continue to resonate in recent additions in the anthropology of death and grief, such as the work of Silverman et al. (2020), who further elaborate on the cultural dynamics of emotions in grief, and the normativities in the relationship between the living and the dead. Contributing to this field of grief research, I discuss in this article the lived experiences of *anticipatory grief*—grief that occurs before, and in anticipation of death. How do people experience and engage with grief for a person who is alive?

Psychological and psychiatric literature has, since the term was coined by Erich Lindemann (1944), long conceived of anticipatory grief as an ‘early start’ to the process of grief work. Grief work, for Freud (1917), referred to the process through which the bereaved subject was supposed to detach itself from the lost object. As grief work became commonly understood to consist of ‘stages’ that the bereaved person passes through (Bowlby 1980; Kübler-Ross 2009), anticipatory grief has been suggested to allow the bereaved to ‘complete’ the grief work quicker (Ponder and Pomeroy 1997; Rando 1983). Rando (1983), for example, has argued that there is an “optimum amount” of anticipatory grief of six to eighteen months for it to have such therapeutic value. Countering such causal and normative thinking in which anticipatory grief is considered to facilitate adaptation to bereavement, Fulton and Gottesman (1980:51; Fulton 2003:348) argue that “anticipatory grief is not simply grief begun in advance; it is different from post-death grief in both duration and form.” Still, this debate, on whether anticipatory grief facilitates, problematizes, or does not relate at all to adaptation to loss after death occurs, has been persistent in clinical psychology and psychiatry, without any clear consensus reached (Moore et al., 2020; Ponder and Pomeroy 1997).

My anthropological approach moves this discussion into a different direction, in that I do not make claims about the relation between anticipatory grief and the grief that follows death, but rather take its ‘anticipatory’ character to reflect the future-oriented character of grief, and particularly to the ways in which future loss influences actions and affects in the present. This temporal dynamic, as I will go on to demonstrate, is twofold. On the one hand, family members, as well as people with dementia themselves, experience losses in the present as the disease progresses. With each change, they grieve for the abilities or aspects of their relationships that have been lost, adapt to the new circumstances, find new ways to relate, and revise their expectations of the trajectory ahead. Gradually, these experiences bring the end of life into view as an increasingly tangible and imminent prospect. On the other hand, then, they become oriented toward the end of life. They anticipate this moment, drawing it into the present, prepare for it practically and emotionally, and grieve for future losses that are expected and loom in the present. Anticipatory grief, then, is temporal and relational, encompassing the experience of losses in the present alongside losses that are still to come.

Further, as a form of grief in life, anticipatory grief infuses interpersonal relations and everyday interactions. Anthropologist Susan Hemer (2017:11) criticizes the concept for theoretically dividing experiences of grief into distinctive categories, countering the presumption that grief before death would be conceptually different from grief after death. However, essential to understanding anticipatory grief is that the person with dementia is present and alive, which means the grieving person continues to engage with them. “To mourn someone who is still alive brings a particular, complicated pain” (Gerrard 2019:208; see also Ponder and Pomeroy 1997), but it also shows that care and (anticipatory) grief are not incompatible. To the contrary, as Kleinman (1988:32) notes, “care becomes an opportunity to grieve,” while, in the words of Garcia (2010:194), “forms of care emerge, not in spite of, but through experiences of loss.” In anticipatory grief, relationships continue, albeit in different form, underscoring that it is a relational phenomenon that takes shape in, emerges from, and produces social interactions (Dragojlovic and Broom 2018). Hence, I suggest that it involves a continuous negotiation between the loss and the continuing relationship.

In my efforts to understand the dialectic in which my interlocutors both experienced the loss of a person and simultaneously maintained meaningful relations with them, it is helpful to better understand how people ‘continue bonds’. Initially coined to account for the ways in which the bereaved can maintain ongoing relationships with a deceased person, a continuing bonds perspective recognizes this ongoing attachment as an integral part of the process of grief—thus countering the classical notion that grief involves undoing this attachment (Klass et al. 1996). This perspective draws attention to the “strategies of connection” (Silverman and Nickman 1996:76) through which the deceased continues to be part of the social reality of the bereaved. These include the memorialization of the deceased through material objects, altars, and site visits, as well as conversations with inner representations of the deceased. Here, I find the concept of continuing bonds useful to provoke recognition of precisely the continuation and adaptation of the relationship in the face of loss. The term ‘continuing’ refers to a relationship that remains after a disruption, which is commonly assumed to be caused by death. However, in the context of dementia, as with many other illnesses, the disruption may be experienced earlier and in a protracted, gradual, or fragmented form. Connecting this perspective to studies in dementia, which have shown approaches to dementia wherein relations are central (Taylor 2017; Moser 2011; Buch 2013, 2015), I illustrate below how family members put great effort in securing a bond with the person with dementia. They continuously reaffirm the relationship, interacting with the person and keeping them present in everyday life.

In this article, I focus on accounts of family members who were concerned with the advancing decline of their relative with dementia, and who were in different ways anticipating further losses toward the end of life. This is not to say that the experiences of those who refrain from anticipation, are to be dismissed, as this too, as Flaherty (2019) notes, can be a temporal commitment and moral project. In the context of dementia, as I will further elaborate below, gradual cognitive and physical decline often provoke the uncanny feeling of “already losing” the person with dementia during the illness trajectory. Moreover, experiences of loss and uncertainty in everyday life with dementia tend to relate to an overdetermined image of the future. The strong negative imaginaries of the future with dementia play an important role in anticipatory grief, whereby the changing capacities of the person with dementia form both a confrontation with loss in the everyday and a confrontation with a preconceived scenario of further decline. While these characteristics may be specific to the context of dementia, the understanding of anticipatory grief that I present in this article also extends to other contexts, particularly other illness trajectories, such as ALS and different forms of cancer, in which patients and their families are confronted with changing capacities and might come to expect future decline and finitude (see, for example, Olson 2014). Pushing this even further, grief is also not limited to illness or the end of life, but can be experienced in relation to other anticipated and experienced ruptures and losses. More generally, then, a nuanced view on anticipatory grief can highlight the intricate layered-ness of grief, loss, and connection—a form of grief of which its emotional force is protracted, and characterized by continuous adaptation to changing circumstances as well as the uncertainty of how the situation will unfold.

The point in writing about loss, then, is not to reproduce an image of dementia as consisting only of deprivation and hurt, but to disentangle and do justice to the nuances in the experiences of my interlocutors, for whom loss and grief were at times overwhelming, but also embedded in relationships of care and connection. Following Lisa Stevenson’s call for an anthropology “that makes room for hesitation” and takes “the uncertain, the confused—that which is not clearly understood—as a legitimate ethnographic object” (2014:2), and with reference to Kierans and Bell’s (2017) plea to cultivate an analytic of ambivalence, I draw attention to how people live and cope with anticipatory grief in everyday life when loss is ongoing, how they relate to future losses in the present, and how they reach out and share time as they navigate the ambiguities of this experience.

## Methodology

The discussion presented is based on 18 months of ethnographic fieldwork in 2014 and 2015, where I took part in everyday life in nursing homes in the Netherlands. This research focused on the end of life with dementia, asking how people with dementia, their family members, and professional caregivers become oriented toward death, and strive to achieve a ‘good’ death. The nursing homes consisted of closed wards with *kleinschalig wonen* (small-scale living) units*,* arranged to resemble households. Through participant observation in these units, I gained insight into everyday life with dementia, the social response to perceived and projected receding capacities, and how the end of life was discussed, negotiated, and experienced. I participated in social activities, drank coffee with residents in the living room, and sat by their bedsides. I was present at medical consultations and accompanied professional caregivers, psychologists, and chaplains in their work with residents. During the afternoons I would often write down field notes in the in-house café on the ward.

Throughout the ethnographic fieldwork in the nursing home, I had informal conversations and conducted semi-structured interviews with professional caregivers, family members, and with people with dementia. I also held forty in-depth interviews with family members in their home setting. Family members were often more at ease when they were at home, and would thus elaborate on sensitive topics such as experiences of loss or their outlook on the end of life. To recruit participants for these interviews I posted a call for participants on the website of the Dutch Alzheimer’s Society (Alzheimer Nederland). Toward the end of my fieldwork, I held a series of three focus groups—one consisting of care workers, one with family members, and one multidisciplinary group of nursing home staff—to discuss insights that had emerged from the field.

Institutionalization at old age is common in the Netherlands, and especially in the context of dementia is often considered inevitable at some point. Nonetheless, institutionalization is a key aspect of negative stereotypical views of a future with dementia, and moving to a nursing home is seen by many as a large and definitive step in the process of decline and loss (van Wijngaarden et al. 2015). In particular, values that are central to the Dutch context, such as autonomy, independence and control, are considered to be at risk when developing dementia. This is also visible in how the future with dementia is often portrayed in popular discourses and media images in the Netherlands. A loss of independence and autonomy are, then, often connected to a perceived loss of dignity (Kennedy 2002; The 2009; van Wijngaarden et al. 2015).^1^

During fieldwork, I usually opened conversations and interviews by asking “What do you find important?” or “Can you tell me your story?” This allowed my interlocutors to start with the subjects they valued, struggled with, or were eager to share, as well as choose where they wanted their story to start. Family members often talked about how their relative with dementia had declined. After having spent an extended period of time in the nursing home, I was able to see for myself how residents’ conditions changed, through which I also gained a more in-depth understanding of the experiences, practices and narratives of family members.

Consent was approached as a process (Dewing 2002; McKillop and Wilkinson 2004) and sought from people with dementia, family members, professional caregivers, and the nursing home management. Access to the nursing homes was granted by the boards of directors and client councils. A letter was sent out to families of all residents to inform them of the research and my presence in the nursing home, as well as their right not to be involved in the project. Consent was further acquired verbally and was verified repeatedly. During recorded interviews, consent was asked on record. Throughout this article, the names and descriptions of the nursing homes and the people who lived, worked, and visited there have been anonymized.

In what follows, I will first elaborate on the ambiguous experiences of family members of people with dementia, who experienced grief even while the person was still alive. Moving on to discuss the ways in which my interlocutors sought to maintain meaningful relations, I underscore the entanglement of loss and connection, showing how both exist parallel to, and may emerge from one another. Then, I highlight the ways in which relationality is enacted through objects and activities that are considered to be of significance to the person with dementia, thus forming also an effort to uphold their subjectivity. I will end by returning to the future-oriented character of anticipatory grief, and show how my interlocutors prepared for the end of life, suggesting that anticipatory grief thus relates to future as well as present losses.

## Ambiguities in Dementia

Many family members referred to dementia in terms of loss, as descending in steps, with intervals between the losses. Patrick, who I introduced above, for example, said: "From her 80th year onwards my mother crumbled in phases. She crumbled, then a status quo. Then another step, status quo. And so on.” Similarly, Irene, whose mother had been living in the nursing home for four years, told me:

“She is changing. The familiarity, the way I used to know her, I feel I am losing that little by little. We see so many changes, and it’s not coming back. Sometimes there is a rare moment, but it all goes gradually. But then, sometimes as steps on a staircase, at once three steps down. It’s a tough process.”

I asked her what kind of moments these “steps” encompassed.

“For example, one of these moments was when I saw that she couldn’t take her medicine anymore. Even though she had always taken that very seriously, she couldn’t oversee that anymore. That was a moment I realized it was going the wrong way.”

Such concrete moments formed a confrontation with particular losses, but also pointed at the larger trajectory of decline and losses yet to come. Decline was a recurring subject of everyday conversation in the nursing home among family members and care staff, as well as in more formal meetings between family and elderly care physicians. By discussing capacities that were deemed lost, family members and care professionals tried to come to an understanding of how the disease was progressing and how they could adapt to the emerging situation. As dementia does not follow a linear trajectory (Moreira 2010; Swallow 2019), loss did not concentrate in a single event but was experienced as a process that occurred in steps or episodes and that could fluctuate. Some days were perceived by family members as better than others, and referred to as “clear moments.” As it cannot be predicted how exactly the disease will manifest, this is an uncertain process, for family members as well as for care workers, who have to adapt to changing care needs, and gerontologists, who may experience difficulties in establishing a diagnosis and a prognosis (Carter, et al., 2015; Leibing 2014; Leibing and Schicktanz 2020; Swallow 2019). Despite these uncertainties, the future trajectory was often narrated with some certainty, projecting further decline and with a prospect of the eventual dying process. In this sense, while glimpses of “clarity” occur, the “downward steps” of loss are, as Irene expressed, also experienced as irreversible and inevitable as they bring the end of life into view. The intervals between losses were often referred to as moments to readjust to the newly unfolding changes; moments of reorientation in which family members sought to understand the lifeworld of the person with dementia, made practical arrangements, and looked for what forms of contact were still possible.

Although my interlocutors tried to adapt to these changes, at some point, many expressed finding it difficult to make contact with their relative with dementia. Els, whose mother had become bedridden and unable to communicate verbally, told me:

“You know, you don’t have your mother anymore. So for example things in my life I’d want to talk about with her, I can’t. In that sense she is completely gone. At first, when you notice she’s losing her memory you try to introduce some memory games, but it becomes less and less. The entire process I have been saying goodbye. You are left with a fragment, a kind of mother, something that remains. Because she is still here.”

The family members I spoke with thus expressed the uncanny feeling that they were losing the person with dementia (“she is completely gone”) while they continued to be a part of their lives (“she is still here”). Researcher and therapist in family studies Pauline Boss (1999, 2011) has termed this “ambiguous loss,” which refers to an experience of loss that is uncertain, partial, and indefinite, yet that may immobilize and “freeze people in place so that they can’t move on with their lives” (1999:20). Further, this experience has been conceptualized in a variety of ways. Sharon Kaufman, for example, has suggested that the categories of life and death can become blurred, and refers to this as a “zone of indistinction” (2005:62) or a “gray zone” (2005:1, 2006:40). She emphasizes that not only the person with dementia themselves may experience a “dislocation of the self,” but also relatives and others involved may encounter a form of dislocation from the person with dementia (2018:556). In so doing, she builds on the work of Janelle Taylor, who observes that people with dementia are often not granted recognition in society, and who advocates a different way of looking at people with dementia, focused on care, so as to involve them “as fully social persons and members of a community” (2008:315).

This ambiguous experience has also been debated extensively in terms of personhood, as early conceptualizations of personhood revolved around cognitive aspects such as memory, control, and rationality, effectively excluding people with dementia (Sweeting and Gilhooly 1990). Challenging the idea that dementia constitutes a loss of personhood, the concept has been reframed to include relationality and the capacities of people with dementia (Kitwood 1993, 1997). Since then, acknowledgement for how personhood is maintained through relations and recognition of the particular care needs and preferences of the person with dementia have become central in these debates (Moser 2011; Buch 2015; Grøn forthcoming). Others have described the ambiguity of absence and presence in terms of “social death” (Sweeting and Gilhooly 1990; see also Králová 2015; Mulkay 1993; Sudnow 1967) or “bare life” (Leibing 2006), which have been used to reveal and challenge the normative frames through which life with dementia is apprehended. These conceptualizations, in other words, reveal the social exclusion of people with dementia from the normative and social frames that make for a meaningful life (Kaufman 2006), and counter dominant biomedical approaches that individualize dementia in the brain and body and that focus solely on the progression of decline (Moser 2011). It thus becomes apparent that dementia can challenge the normal order of things at the end of life. Trying to bring her own experience into words, Els said:

“It is a very strange sadness. It’s grief. Because she is there, but also isn’t. That’s what is so difficult. I visit every week, and then I am very happy to have come, because it makes her so happy. That is wonderful. But well, I don’t have my mother anymore.”

Tears began to well up in her eyes as she continued:

“Actually, it is terrible. So many emotions, but you know you have to keep going. You keep it in to keep it manageable. I try not to give in to that too much, not to think about the sadness of it all. And certainly not to treat her as if she is not there, to the contrary. I want to keep alive what we still have. And when that succeeds, it makes me happy.”

Hence, while Boss has theorized that ambiguous loss ‘immobilizes’ and prevents people from adjusting (1999), I rather show that in the experience of ambiguity, family members of people with dementia experience grief and simultaneously find ways to cope with this, to continue life by searching for ways to maintain meaningful relations.

## Moments of (Dis)connection

As I described above, Patrick told me he was aware of the gradual, “stepwise” decline his mother went through and struggled with the ambiguous sense that he was already grieving while she was still alive. On his Sunday afternoon visits, he always brought a bottle of white wine. At that time, Patrick’s mother was bedridden and unable to verbally communicate. In the wine, he seemed to have found a way to make contact. “I felt the pain of seeing her declining,” he continued, “but you could also see that she was still able to enjoy certain things. So I brought a good bottle for the two of us, to enjoy together. She loved a nice Sauvignon. I would let her smell the wine first. And then we would just finish the bottle together.” Even though at times it felt as if he already lost her, Patrick was able to recognize and confirm his mother’s presence and their relationship through a meaningful object—the wine. He put great effort in finding ways to sustain the relationship between them, creating moments they could share that make a continuing bond possible. In his experience of disconnection, Patrick found moments of connection.

Through such efforts of care, family members negotiated the ambiguity of absence and presence they experienced. Repeatedly, they looked for opportunities in light of what is lost and what is still possible. For example, in the second month of my fieldwork, Paul moved into the nursing home. He was in his mid-eighties and had been an organ player and teacher. His wife, Annemiek, sang in a church choir. Music played an important part in their lives. Paul was a very gentle man but was often restless and demanded much attention, which he expressed by yelling at others every other minute, “Hey, you! Come over here! Sit down here,” pointing at the chair next to him. Annemiek had compiled a playlist on an iPod with music and sounds that she considered part of his biography. The motivation for this was twofold: it allowed Paul to listen to music that he enjoyed, while it was also an attempt to calm him down.^2^

Annemiek visited him several times per week. She said: “You know, every time I am on my way here I feel a certain anxiety, a tension, and it is because I never know in what mood he will be.” One afternoon, she brought a small keyboard with her and played it in the living room, while Paul and other residents listened. After a couple of songs she placed the keyboard in front of Paul. In his attempt to play, his fingers moved stiffly across the keys and created a fragmented melody. Annemiek turned toward me with a sad look and said “he is not as good as he used to be.” Annemiek, like many family members, was concerned with how he was changing and the uncertainties this involved. And like many others, she tried to make the best of the situation by seeking ways to connect. However, while she had brought the keyboard to generate a way of being together that reflected activities they used to share, Paul’s inability to play as he used to also made his receding capacities visible: a confrontation with loss in seeing what was no longer possible.

Such a sense of coinciding connection and disconnection also came to the fore when Annemiek told me that she and Paul used to listen to Radio 4, a channel for classical music. Now that Paul lived in the nursing home, she had asked the care staff to turn on the radio in his room. Oftentimes when she was listening to the radio at home, she wondered: “Does he have the radio on at this moment? Is he also listening to this piece?” Simultaneously listening to the same music, created the idea of being connected while apart. For Annemiek and Paul, then, music allowed the bond between them to continue in several ways: as something they could share, listening and playing together, or that made Annemiek relate to Paul when they were away from each other. But it also painfully underscored the several losses.

Gerda, too, was confronted with what was no longer possible precisely in her attempt to connect with her mother who lived in a nursing home. She told me:

“I had bought peas. We used to shuck them together, so when I was at the market I thought, oh peas, yes that will be nice, like we used to do. But when I gave them to her she had no idea what to do with them. Even after I showed her, she couldn’t.”

Similar to Patrick, who brought wine, and Annemiek, through music, Gerda brought peas with the intention to create a shared activity that reflected their past, and continuing relation. However, while she looked for a way to maintain a connection through a meaningful object (the peas), the seeming failure of this attempt made the loss tangible. Anticipatory grief and meaningful relations, or loss and connection, then, do not stand in dual opposition to each other. Rather, they fit together and can emerge simultaneously or from each other.

In this way, the relationship with the person with dementia can oscillate between connection and disconnection. Family members continuously readjust how to connect, as they care for, and move along with the process of dementia. They look for what the person is still capable of, and what forms of connection are still possible. Inspired by the work of Elinor Fuchs (2005, in Taylor 2008), who referred to this as “stills,” Janelle Taylor (2008:316) writes that “the ‘stills’ are paralleled by ‘firsts’.” An attunement to “stills,” in other words, also involves moments at which it becomes clear for the first time that the person with dementia is unable to do something she was capable of doing earlier in life. Both “firsts” and “stills” can be painful and joyful (Taylor 2008). For Gerda, the first time she realized her mother was unable to shuck peas came as a confrontation with loss. Her sister, Monica, shared a very different experience. She told me about a dancing afternoon organized in the nursing home, where, for the first time in her life she had danced with her mother. In some cases, then, “firsts” referred to new ways of making contact. It is thus vital to understand that “grief can also constitute ways of relating, of giving, and of receiving” (Danely 2014:105), and be seen as a constant negotiation between moments of loss and joy.

## Maintaining (Inter)subjectivity

In these moments of connection and disconnection, wherein my interlocutors sought to maintain their relationship with the person with dementia, not only the intersubjective bond was considered to be at stake and re-emphasized, but also the subjectivity of the person with dementia. This resonates with scholarship that has in recent years focused on various practices and dynamics of care through which the subjectivity of the person with dementia is maintained (Buch 2015; Seaman 2020; Taylor 2008). In her work on a closed dementia ward in Norway, Ingunn Moser presents what she calls a “relational approach to life and dementia” (2011:708), demonstrating how professional caregivers would maintain the person with dementia in relations through practices involving music and food. Similarly, in her study of home care for older adults in the United States, Elana Buch (2013, 2018) explores how the embodied practices of care workers may sustain the personhood, ways of life, and sense of self of the elders. Also primarily working in the United States, Janelle Taylor (2017) writes on dementia and friendship, and suggests that friends, family, and health professionals of people with dementia are actively involved in moral and social experimentation with art and relationships, so as to maintain connections and personhood. These studies have thus elucidated, in various ways, how care practices for older adults and people with dementia, may be oriented toward sustaining their subjectivity and relationality.

In a similar vein, I observed that family members placed much emphasis on who the person with dementia was. They sought to confirm continuity, that this person was “still here.” Patrick told me that, after his father had passed away, his mother started studying and traveling. He described her as a “*petite madame,* like a *Française*” who had always loved a good glass of wine. Hence, he linked the wine he brought on his visits specifically to his mother’s person. It represented an aspect of her life in the past that she valued and enjoyed. Bringing a bottle of wine with him on his visits to his mother in the nursing home, then, was not only a way to create an activity together and maintain the bond between them. Doing so also confirmed the person she had been and still was.

In a different case, I brought Ms. Kortrijk to the hairdresser, which was located downstairs in the nursing home complex. While the hairdresser washed Ms. Kortrijk’s hair and put hair curlers in, she told me that she knew Ms. Kortrijk well. “How often people come here differs per person,” she said. “Some people I see every week, others only once a month or even less. It very much depends on the family.” In Ms. Kortrijk’s case, her family had agreed with the nursing home that she would be brought to the hairdresser every other week. When I later spoke to Ms. Kortrijk’s sister, she brought this up herself, and said:

She always looks presentable, which I find very important. These are things she has always cared about. She was a real fashion doll, later she became a model. She always looked amazing, with high heels, tightly dressed, and her hair in tip-top shape from the hairdresser. If you look at pictures of her, you will see how she was, really a beautiful woman. So we find it important to continue this aspect of her.

In this sense, family members were actively involved in cultivating situations through which to nurture the activities, objects, and environment that had been of importance to the person with dementia in the past. The objects and practices through which they did so, were thus also considered significant to the relationship and the person, as carrying the potential of connection and to uphold subjectivity. In many cases, this involved bringing material objects that had been of significance in the past, into the present, as in Patrick’s case with the wine.

Just as family members found it important to share moments with the person with dementia, and were involved in the maintenance of subjectivity, people with dementia themselves were also concerned with upholding a sense of self when they felt this was slipping away from them. Bob, for example, a 60 year old man who had dementia and lived at home with his wife, was passionate about old cars, and was helping a friend to fix his 1959 Mercedes. Together, they were taking apart and reassembling the vehicle. “What I find difficult,” Bob said, was that “I used to just throw all those parts in a box, and I would easily remember what it contained.” Now, his friend had prepared and labeled small storage boxes for all the different parts and bolts. As they were reassembling the car, his friend would hand Bob the right parts, allowing Bob to exert his knowledge of how to assemble them and get the car running again. “I told him, if you mess with these boxes, that car is never going to be back in one piece.” While writing, typing, reading, making phone calls, had all become increasingly difficult, his ability to work with mechanics largely remained, and so he proudly told me that he had recently fixed a neighbor’s dishwasher. “I then take pictures of it to remind myself how to put it back together.” He was aware of the gradual diminishing of certain abilities, and knew that he had become unable to do things by himself as he used to. “My handwriting was always terrible, but now I can’t even read it myself,” he commented. Yet together with others he was able to practice his hobbies and to continue to exercise his long-standing insights in mechanics. These were important moments for Bob, which allowed him to keep his world going through practices and objects that mattered to him, in relation to others.

Family members and people with dementia themselves thus responded to a perceived loss through objects and practices that were considered important to the person with dementia. Also professional caregivers in the nursing home, I observed, continuously sought to provide care in ways that matched with each particular resident. They often insisted on explicitly addressing people by their name, and it was quite common for them to attribute choices they made in providing care to the preferences of residents, for example by helping a resident who was “not a morning person” to get out of bed only after the others had been helped.

There may be a risk of fixating the person with dementia by attempting to maintain subjectivity in this way. Hilde Lindemann (2014) describes how the maintenance of personhood in interaction with others can occur through holding on and letting go of aspects of their identity. She emphasizes that being “held” in personhood may not be a positive experience but rather may be a site of friction between the different actors involved—an insight that rings especially true when family members hold on to aspects of the identity of the person with dementia that may have changed through the course of the illness. In some cases, as described above, my interlocutors experienced loss not just when the person became unable to participate in a certain activity, but when this inability represented a loss of their supposed identity. Still, the efforts of family members and people with dementia themselves to uphold a sense of identity, even if sometimes faltering, may be understood as attempts to cope with the ambiguous experiences of loss and connection. As I will describe next, these experiences are not only a response to present changes, but also anticipatory—as in relating to, and preparing for, the future.

## Grieving in Anticipation

Patrick emphasized that the bottle of white wine he brought on his weekly visits to his mother in the nursing home should be “a good one, since every glass could be her last one. I even stopped buying wine at the nursing home restaurant. I don’t want her to have bad wine as her last.” His preoccupation with the quality of the wine reflected his awareness that his mother’s death was approaching, even if it was uncertain when this would be. His efforts to maintain a connection, then, were not only framed by the losses he had already experienced—the wine being one of the few things they could still enjoy together—but also by the *anticipation* of losses to come. “Emotionally, you want to keep your mother with you as long as possible,” Patrick said, “and I can see that she was still able to experience pleasure. But on the other hand, the nursing home, as I see it, is the last station. I have been calling it death’s waiting room for a reason.” Like Patrick, many of my interlocutors expected further degeneration, and the eventual death of their relative with dementia. Even as they found moments of connection and enjoyment, their experiences were also colored by this looming horizon. Their grief, then, related both to present and future losses. It indeed involves anticipation, in that it pertains to the expectation of, and preparation for, losses to come. And even as many of them felt they had “already lost” their relative with dementia, death increasingly came into view as an imminent prospect, and as an object of grief in the present.

I suggest that the anticipatory character of this grief lies in that it relates to a projected future with tangible implications in the present. I build here on anthropological explorations of the future that have shown that past, present and future are interwoven and mutually constituted (Mattingly 2014; Munn 1992; Bryant and Knight 2019). Anticipation, as I have elaborated elsewhere (Lemos Dekker 2021), can be understood as a temporal orientation that draws the future into the present, while also keeping it at bay, as being “not yet.” It involves the creation of future imaginaries, which may then inform and legitimize actions and affects in the present (Adams et al. 2009; Bryant and Knight 2019) and is grounded in lived experiences (Stephan and Flaherty 2019). Anticipatory grief, then, is to grieve a loss that is still to occur; to already feel it, prepare for it, and act upon it. Simultaneously, these losses that are expected to happen, are folded into the experience of losses that occur in the present or that have already happened (see also Olson 2014). The “stills” and “firsts” that, as I described above, are part of the experience of “going down a staircase,” whereby each step is accompanied by uncertainties that may trigger grief, also point at changes that are yet to come. The experience of grief becomes iterative, accumulative, and prelusive, all at once.

Many of my interlocutors indicated that they were preparing themselves, in one way or another, for the expected death of their relative with dementia. This was also the case in Sonja and Ina’s situation. Their mother had been living in the nursing home for a number of years and, at the time that I interviewed them, she was bedridden. The sisters took turns in visiting her, and were additionally involved in volunteering at the nursing home, by making pancakes for the entire unit every few weeks. When I interviewed Sonja for the first time, she admitted to being quite nervous for our conversation, finding it difficult to talk about her mother without becoming “too emotional.” It could often be difficult to find a quiet spot to talk on the ward, so from time to time I could use the small office of the nursing home manager for interviews. Being able to close the door behind us, allowed for more privacy and to talk more at ease. During our conversation, Sonja reflected on the different ways in which she was preparing for her mother’s death. “I want to give a speech at my mother’s cremation. But when I sit down, only tears come up and I get nothing on paper. So now I think, maybe I will think of something once she has passed away.” Sonja and Ina had been looking through pictures of their mother for the funeral card, had selected clothes they thought their mother should be dressed in at the funeral, and had discussed what to do with her ashes afterward. “All these things to think about,” Sonja sighed, “but perhaps it’s easier now. So that, when the moment is there, we can say goodbye and just be sad, not having to arrange all this. Because we can do all of these things in advance.” Sonja pointed at being able to make arrangements for her mother’s funeral, already weeks and months before she eventually passed away. This involved making meaningful preparations for the end of life, as well as anticipating the loss itself. “I really like the song Mama, by Il Divo, and we want to play this at the cremation ceremony,” Sonja explained with watery eyes. “So now I am already practicing to listen to it without bursting into tears. I often listen to it while making dinner, but then I can’t hold myself and start to sob.”

Two weeks after this conversation with Sonja, I spoke to her sister Ina, about their mother’s end of life. She said that she thought she was ready for it, knowing that “it will happen anyway. It’s like a dark cloud hanging over me.” She continued:

“I take this possibility into account in everything I do. A few weeks ago, I took a little vacation, but we decided not to go too far. Because what if we get called and would have to get back here in the middle of the night? Then we wouldn’t be able to return on time. It’s very tiring, to constantly have to take this into account. I do everything I would normally do, but it’s always on my mind. It’s like being in a waiting room.”

Like many other interlocutors, Sonja and Ina were attuned to their mother’s approaching death. Being moved by this anticipated loss, then, also meant being moved *toward* it (see also Ahmed 2004). To anticipate is directional, oriented toward a particular future. In preparing for this moment, by making a funeral card, trying to write a speech, and making decisions in everyday life, or, as in Patrick’s case, by bringing a good bottle of wine because each time it could be the last one, they acted upon a loss that had not occurred yet. Also the experience of waiting, of being “on hold,” which many of my interlocutors brought up, shows how they were focused on a moment that had not arrived yet but which they knew was going to occur. Further, anticipation is affective, as others have pointed out (Ahmed 2004; Adams et al. 2009). After my initial conversations with Sonja and Ina, it would take another couple of months before their mother eventually passed away. Nevertheless, in their experience, this future moment had been very near and concrete, almost as if it was a reality already. It involved hurt, sadness, and frustration. And at the same time, Ina also indicated that she was thankful for having the time to prepare for her mother’s death, being able “to just be with her, to touch her.”

## Conclusion

In this article, I have shown that, in the experiences my interlocutors shared with me, loss and connection are continuously interwoven, that efforts to maintain a relationship with the person with dementia in the face of loss are also aimed at sustaining their subjectivity, and that anticipatory grief pertains to accumulating losses experienced in the present together with expected, future losses.

With this, I have asked what we can learn from grief in life, and how to account for this anticipatory grief anthropologically? An anthropological approach to anticipatory grief can reveal the nuanced and equivocal character of experiences of illness and at the end of life. From my interlocutors’ experiences, it becomes apparent how they struggled with this ambiguity. I was repeatedly struck by their expressions of having “already lost” the person with dementia, but was impressed equally so by their persistence in finding ways to care and connect with the person and uphold their subjectivity. For my interlocutors, who cared for a family member with dementia, or who had dementia themselves, this was indeed an experience of grief. But it was also one of contact, togetherness and potentiality. In some instances, the search for connection precisely formed a confrontation with loss, while at the same time implied that there was still room for adaptation and negotiation. This experience shows that loss and connection are not opposites, but revolve around one another, whereby one may emerge from the other and both may combine into a single experience. Without reinforcing stereotypical representations of dementia, I have sought to do justice to both the grief—it’s loss, sadness, and frustration—and the joy and creativity with which my interlocutors found ways of being together and going through life. This entanglement, I suggest, is essential to understanding anticipatory grief.

Anticipatory grief is by no means limited to dementia. At the same time, reflecting on dementia as I have done here, reveals some of the workings of anticipatory grief, and may be suggestive to how it develops in other circumstances. Grief can take place in relation to losses that occurred in the past and that are expected to happen in the future. This may involve changes in selves and relationships, as well as the (anticipated) grief for the loss of a person. Preparing for loss can take many different forms, which may include planning activities, adapting expectations, and recalling past experiences. The experiences and practices of my interlocutors show how anticipatory grief can play an important role in preparing for changes that are yet to come. I have shown that understanding the grief for a loss that is yet to occur, requires bringing into view how present and future relate to one another. Grief for what has been lost, and grief for what will be lost, then become woven into a single experience.

In these everyday negotiations of loss and connection, future and present thus become entwined, in that these experiences relate both the current situation and to expected changes. In anticipatory grief, then, the loss itself is both experienced and projected. The end of life did not suddenly or unexpectedly present itself to my interlocutors, but gradually came into view as dementia progressed. While dementia can be a confrontation with loss, the possibility to enjoy life and to create shared, meaningful moments also remains. And whereas certain situations may seem unbearable beforehand, once they occur people may learn to cope with them (Pols and Limburg 2016). Hence, as the condition of the person with dementia changed, my interlocutors time and again sought new ways to adapt and secure the bond between them. And even though it would remain uncertain how exactly the person with dementia would change or how fast the disease would progress, they were already looking ahead for what might then still be possible. “That means, doing everything that’s possible. Looking at old photographs, bringing sweets and fruits she loved, letting her enjoy the wine,” Patrick said, showing that, infused in loss, was a commitment to presence.

## Footnotes

1 Autonomy and control play an important role in the framing of a good death in the Netherlands. The legal possibility of euthanasia, in particular, is indicative of the centrality of these values in Dutch discourses and practices at the end of life. In some cases, a reorientation to the end of life with dementia involves exploring the possibilities of euthanasia. I have written about such reorientation toward the future through a euthanasia request elsewhere (see Lemos Dekker 2021).

2 While the effect of music on people with dementia is beyond the scope of this article, there have been lively public and academic debates on music as a way of making contact with, and stimulating the capacities of people with dementia. See, for example, McDermott et al. (2014).

## References

[CR1] Adams Vincanne, Murphy Michelle, Clarke Adele E (2009). Anticipation: Technoscience, Life, Affect, Temporality. Subjectivity.

[CR2] Ahmed Sara (2004). The Cultural Politics of Emotion.

[CR4] Blandin, Kesstan, and Renee Pepin 2015 Dementia Grief: A Theoretical Model of a Unique Grief Experience. Dementia. 16(1): 67–78 10.1177/1471301215581081 PMC4853283 25883036

[CR5] Boss Pauline (1999). Ambiguous Loss: Learning to Live with Unresolved Grief.

[CR6] Boss, Pauline 2011 Loving Someone Who Has Dementia: How to Find Hope While Coping with Stress and Grief San Fransisco: Jossey-Bass. Kindle edition

[CR7] Bowlby, John 1980 Attachment and Loss, Volume III: Loss Sadness and Depression New York: Basic Books

[CR8] Bryant Rebecca, Knight Daniel M (2019). The Anthropology of the Future.

[CR9] Buch Elana D (2013). Senses of Care: Embodying Inequality and Sustaining Personhood in the Home Care of Older Adults in Chicago. American Ethnologist.

[CR10] Buch Elana D (2015). Anthropology of Aging and Care. Annual Review of Anthropology.

[CR11] Buch Elana D (2018). Inequalities of Aging: Paradoxes of Independence in American Home Care.

[CR12] Carter Gillian, van der Steen Jenny T, Galway Karen, Brazil Kevin (2015). General Practitioners’ Perceptions of the Barriers and Solutions to Good-Quality Palliative Care in Dementia. Dementia.

[CR13] Danely Jason (2014). Aging and Loss: Mourning and Maturity in Contemporary Japan.

[CR14] Dewing Jan (2002). From Ritual to Relationship: A Person-Centered Approach to Consent in Qualitative Research with Older People Who Have a Dementia. Dementia.

[CR15] Dragojlovic Ana, Broom Alex (2018). Bodies and Suffering: Emotions and Relations of Care.

[CR16] Driessen Annelieke (2018). Pleasure and Dementia: On Becoming an Appreciating Subject. The Cambridge Journal of Anthropology.

[CR17] Flaherty Devin (2019). ’Takin’ It One Day at a Time’: (Not) Anticipating as Moral Project. The Cambridge Journal of Anthropology.

[CR18] Freud, Sigmund 2005 [1917] On Murder, Mourning and Melancholia London: Penguin Books

[CR19] Fulton Robert (2003). Anticipatory Mourning: A Critique of the Concept. Mortality.

[CR20] Fulton Robert, Gottesman David J (1980). Anticipatory Grief: A Psychosocial Concept reconsidered. British Journal of Psychiatry.

[CR21] Garcia Angela (2010). The Pastoral Clinic Addiction and Dispossession Along the Rio Grande.

[CR22] Gerrard Nicci (2019). What Dementia Teaches Us About Love.

[CR23] Grøn Lone, Mattingly Cheryl (2018). In Search of the Good Old Life: Ontological Breakdown and Responsive Hope at the Margins of Life. Death Studies.

[CR24] Grøn, Lone Forthcoming Yeah … Yeah. Imagistic Signatures and Responsive Care at a Danish Dementia Ward. *In* Imagistic Care. Growing Old in a Precarious World C. Mattingly, and L. Grøn, eds. Fordham University Press

[CR25] Hemer Susan R (2017). Preparing for Death: Care, Anticipatory Grief and Social Death in Lihir, Papua New Guinea. Mortality.

[CR26] Hertz, Robert 2004 [1907] A Contribution to the Study of the Collective Representation of Death. *In* Death, Mourning, and Burial: A Cross-Cultural Reader A.C.G.M. Robben, ed. Malden: Blackwell Publishing

[CR27] Howarth Glennys (2007). Death and Dying: A Sociological Introduction.

[CR28] Huntington Richard, Metcalf Peter (1979). Celebrations of Death: The Anthropology of Mortuary Ritual.

[CR29] Jeong Jong-min (2020). The Affective Creativity of a Couple in Dementia Care. Culture, Medicine, and Psychiatry.

[CR30] Kaufman Sharon R (2005). …And a Time to Die: How American Hospitals Shape the End of Life.

[CR31] Kaufman Sharon R, Leibing A, Cohen L (2006). Dementia-Near-Death and "Life Itself". Thinking About Dementia: Culture, Loss, and the Anthropology of Senility.

[CR32] Kaufman Sharon R (2018). "Losing My Self": A Poet's Ironies and a Daughter's Reflections on Dementia. Perspectives in Biology and Medicine.

[CR33] Kennedy James (2002). Een weloverwogen dood: euthanasie in Nederland.

[CR34] Kierans, Ciara, and Kirsten Bell 2017 Cultivating Ambivalence: Some Methodological Considerations for Anthropology. HAU: Journal of Ethnographic Theory 7(2). 10.14318/hau7.2.006

[CR35] Kitwood Tom (1993). Person and Process in Dementia. International Journal of Geriatric Psychiatry.

[CR36] Kitwood Tom (1997). Dementia Reconsidered: The Person Comes First.

[CR37] Klass Dennis, Silverman Phyllis R, Nickman Steven L (1996). Continuing Bonds: New Understandings of Grief.

[CR38] Kleinman Arthur (1988). The Illness Narratives: Suffering, Healing, And the Human Condition.

[CR39] Králová Jana (2015). What is Social Death?. Contemporary Social Science.

[CR40] Kübler-Ross, Elisabeth 2009 [1970] On Death and Dying: What the Dying Have to Teach Doctors, Nurses, Clergy and Their Own Families London: Routledge.

[CR41] Large Samantha, Slinger Richard (2015). Grief in Caregivers of Persons with Alzheimer’s Disease and Related Dementia: A Qualitative Synthesis. Dementia.

[CR42] Leibing Annette, Leibing A, Cohen L (2006). Divided Gazes: Alzheimer's Disease, the Person Within, and Death in Life. Thinking About Dementia: Culture, Loss, and the Anthropology of Senility.

[CR43] Leibing Annette (2014). The Earlier the Better: Alzheimer’s Prevention, Early Detection, and the Quest for Pharmacological Interventions. Culture, Medicine, and Psychiatry.

[CR44] Leibing Annette, Schicktanz Silke (2020). Preventing Dementia? Critical Perspectives on a New Paradigm of Preparing for Old Age.

[CR78] Lemos Dekker Natashe (2021). Anticipating an Unwanted Future: Euthanasia and Dementia in the Netherlands. Journal of the Royal Anthropological Institute.

[CR45] Lindauer Allison, Harvath Theresa A (2014). Pre-death Grief in the Context of Dementia Caregiving: A Concept Analysis. Journal of Advanced Nursing.

[CR46] Lindemann Erich (1944). Symptomatology and Management of Acute Grief. American Journal of Psychiatry.

[CR47] Lindemann Hilde (2014). Holding On and Letting Go: The Social Practice of Personal Identities.

[CR48] Mattingly Cheryl (2014). Moral Laboratories: Family Peril and the Struggle for a Good Life.

[CR49] McDermott Orii, Orrell Martin, Ridder Hanne Mette (2014). The Importance of Music for People with Dementia: The Perspectives of People with Dementia, Family Carers, Staff and Music Therapists. Aging and Mental Health.

[CR50] Mckillop James, Wilkinson Heather (2004). Make it Easy on Yourself: Advice to Researchers from Someone with Dementia on Being Interviewed. Dementia.

[CR51] Moore Kirsten J, Moore Sophie Crawley, Vickerstaff Victoria, Cooper Claudia, King Michael, Sampson Elizabeth L (2020). Is Preparation for End of Life Associated with Pre-death Grief in Caregivers of People with Dementia?. International Psychogeriatrics.

[CR52] Moreira, Tiago 2010 Now or Later? Individual Disease and Care Collectives in the Memory Clinic. *In* Care in Practice: On Tinkering in Clinics, Homes and Farms A. Mol, I. Moser, and J. Pols, eds. Bielefeld: Transcript

[CR53] Moser Ingunn (2011). Dementia and the Limits to Life: Anthropological Sensibilities, STS Interferences, and Possibilities for Action in Care. Science, Technology and Human Values.

[CR54] Mulkay Michael, Clark D (1993). Social Death in Britain. The Sociology of Death.

[CR55] Munn Nancy D (1992). The Cultural Anthropology of Time: A Critical Essay. Annual Review of Anthropology.

[CR56] Olson RE (2014). Indefinite Loss: The Experiences of Carers of a Spouse with Cancer. European Journal of Cancer Care.

[CR57] Peacock Shelley, Bayly Melanie, Gibson Kirstian, Holtslander Lorraine, Thompson Genevieve, O’Connell Megan (2018). The Bereavement Experience of Spousal Caregivers to Persons with Dementia: Reclaiming Self. Dementia.

[CR58] Pols Jeannette, Limburg Sarah (2016). A Matter of Taste? Quality of Life in Day-to-Day Living with ALS and a Feeding Tube. Culture, Medicine and Psychiatry.

[CR59] Ponder Rebecca J, Pomeroy Elizabeth C (1997). The Grief of Caregivers: How Pervasive Is It?. Journal of Gerontological Social Work.

[CR60] Radcliffe-Brown, A.R 2004 [1922] The Andaman Islanders. In Death, Mourning, and Burial: A Cross-cultural Reader. A.G.C.M. Robben, eds., pp. 151–155. Malden: Blackwell Publishing.

[CR61] Rando Therese A (1983). An Investigation of Grief and Adaptation in Parents Whose Children Have Died from Cancer. Journal of Pediatric Psychology.

[CR62] Robben, Antonius C.G.M., ed 2004 Death, Mourning, and Burial: A Cross-Cultural Reader Malden: Blackwell Publishing.

[CR63] Rosaldo, Renato 2004 [1989] Grief and a Headhunter’s Rage. In Death, Mourning, and Burial: A Cross-cultural Reader. A.G.C.M. Robben, ed. Malden: Blackwell Publishing.

[CR64] Rosaldo Renato (2014). The Day of Shelly′s Death: The Poetry and Ethnography of Grief.

[CR65] Scheper-Hughes Nancy (1993). Death Without Weeping: The Violence of Everyday Life in Brazil.

[CR66] Seaman Aaron T (2020). “Like He’s a Kid”: Relationality, Family Caregiving, and Alzheimer’s Disease. Medical Anthropology.

[CR67] Shohet Merav (2018). Two Deaths and a Funeral: Ritual Inscriptions’ Affordances for Mourning and Moral Personhood in Vietnam. American Ethnologist.

[CR3] Silverman Gila S, Baroiller Aurélien, Hemer Susan R (2020). Culture and Grief: Ethnographic Perspectives on Ritual, Relationships and Remembering. Death Studies.

[CR68] Silverman, Phyllis R., and Steven L. Nickman 1996 Children's Construction of Their Dead Parents. In Continuing Bonds: New Understandings of Grief. D. Klass, P.R. Silverman, and S.L. Nickman, eds. New York: Routledge.

[CR79] Stephan Christopher, Flaherty Devin (2019). Introduction. Experiencing Anticipation: Anthropological Perspectives. The Cambridge Journal of Anthropology.

[CR69] Stevenson Lisa (2014). Life Beside Itself: Imagining Care in the Canadian Arctic.

[CR70] Sudnow David (1967). Passing on: The Social Organization of Dying.

[CR71] Swallow Julia (2019). Constructing Classification Boundaries in the Memory Clinic: Negotiating Risk and Uncertainty in Constituting Mild Cognitive Impairment. Sociology of Health and Illness.

[CR72] Sweeting Helen N, Gilhooly Mary L.M. (1990). Anticipatory Grief: A Review. Social Science and Medicine.

[CR73] Taylor Janelle S (2008). On Recognition, Caring, and Dementia. Medical Anthropology Quarterly.

[CR74] Taylor Janelle S (2017). Engaging with Dementia: Moral Experiments in Art and Friendship. Cult Med Psychiatry.

[CR75] The Anne-Mei (2009). Verlossers naast God: dokters en euthanasie in Nederland.

[CR76] van Gennep, Arnold 2004 [1909] The Rites of Passage. *In* Death, Mourning, and Burial: A Cross-Cultural Reader. A.C.G.M. Robben, ed. Malden: Blackwell Publishing

[CR77] van Wijngaarden Els, Leget Carlo, Goossensen Anne (2015). Ready to Give Up on Life: The Lived Experience of Elderly People Who Feel Life is Completed and No Longer Worth Living. Social Science and Medicine.

